# Brominated Thiophenes as Precursors in the Preparation of Brominated and Arylated Anthraquinones

**DOI:** 10.3390/molecules14031013

**Published:** 2009-03-04

**Authors:** Thies Thiemann, Yasuko Tanaka, Jesus Iniesta

**Affiliations:** 1 Interdisciplinary Graduate School of Engineering Sciences Kyushu University, 6-1, Kasuga-koh-en, Kasuga-shi, Fukuoka 816-8580, Japan; 2 Institute of Materials Chemistry and Engineering, Kyushu University, 6-1, Kasuga-koh-en, Kasuga-shi, Fukuoka 816-8580, Japan; 3 Department of Physical Chemistry, University of Alicante, E-03080 Alicante, Spain; E-mail: jesus.iniesta@ua.es (J.I.)

**Keywords:** Anthraquinone, Oxidative cycloaddition, Suzuki cross coupling, UV spectroscopy.

## Abstract

Brominated anthraquinones can be synthesized directly from bromothiophenes when these are reacted with 1,4-naphthoquinones in the presence of *meta-*chloroperoxy-benzoic acid. The bromoanthraquinones are versatile building blocks in the preparation of arylated anthraquinones and of extended *π*-systems with interspersed anthraquinone units.

## 1. Introduction

**Figure 1:**
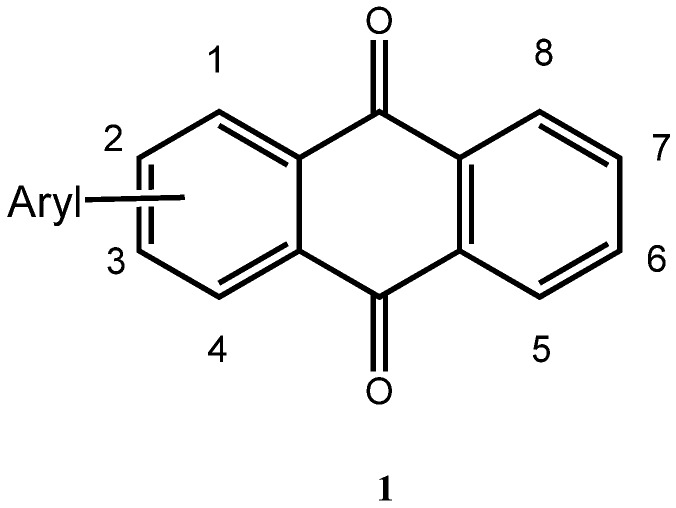
General Structure of arylated Anthraquinones.

Arylated anthraquinones **1** (Figure 1) have elicited interest in Physical Organic Chemistry [1,2] due to the interaction of the attached aryl groups with the *π*-system of the anthraquinone core, as evidenced in the corresponding UV and luminescence spectra [4,5], in the redox behavior of the molecules [2,3], and their NMR shift values. Specifically, the interaction of the substituents on the anthraquinone C=O function has been subjected to investigation [1]. In practical applications, arylated anthraquinones have also been used as stabilizers of light-modulating fluids such as of fluids comprised of liquid polybenzyltoluenes [6]. Our interest in these molecules is in the study of their electrochemical behavior. In the following, a new direct preparation of arylated anthraquinones from bromothiophenes is presented.

**Scheme 1:**
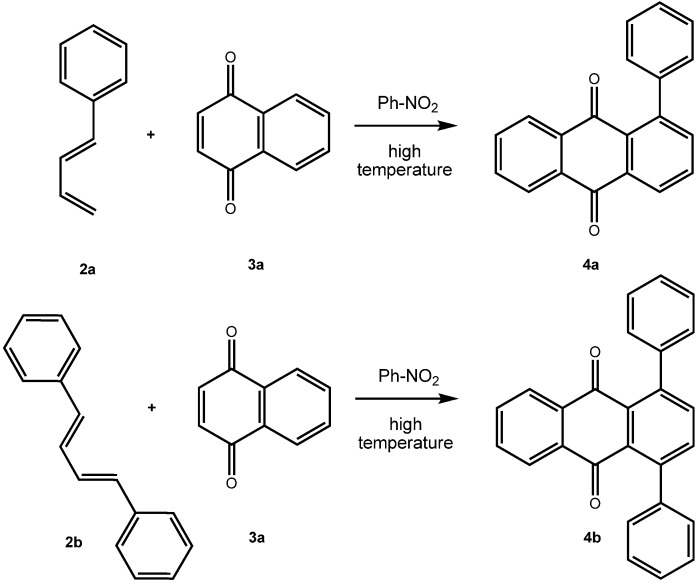
Aryl substituted anthraquinones by [4+2]-cycloaddition reaction [7,8].

**Scheme 2:**
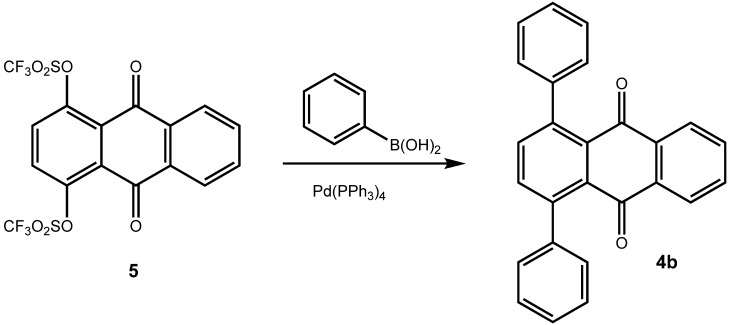
1,4-Diphenylanthraquinone by Suzuki-type coupling with aryl triflates [2].

A number of synthetic routes to arylated anthraquinones are known. It has been shown by Bergmann *et al*. [7,8] that [4+2]-cycloaddition reactions of phenylbutadienes **2** with either 1,4-naphthoquinone (**3a**) or with *p*-benzoquinone give 1-phenylanthraquinone (**4a**) and 1,4-diphenylanthraquinone (**4b**) (from 1,4-naphthoquinone) and 1,5-diphenylanthraquinone and 1,4,5,8-tetraphenylanthraquinone (from *p*-benzoquinone), respectively (Scheme 1). The Diels-Alder approach has also been used for the synthesis of arenoanthraquinones such as of benz[a]anthracene-7,12-diones [9]. For the preparation of 1,4-diarylanthraquinones, Gautrot *et al*. [2] started from 1,4-dihydroxy-9,10-anthraquinone, which was transformed into its bistriflate **5** [3] and subsequently subjected to coupling reaction with arylboronic acids (Scheme 2) [2].

Coupling reactions have also been carried out with 1-diazoanthraquinone, which was prepared from the corresponding 1-aminoanthraquinone [10]. In order to have a versatile strategy to prepare aryl substituted anthraquinones in hand, we wanted to use haloanthraquinones as key intermediates, which we could subsequently transform into the target compounds by Suzuki cross coupling reactions. Again, preparative routes to haloanthraquinones are known. Thus, Battegay and Claudin prepared a number of dibromoanthraquinones from the corresponding diaminoanthraquinones by Sandmeyer reactions [11] and sulfonic acid functionalities could also be transformed to bromo substituents at elevated temperatures [11]. 

**Scheme 3:**
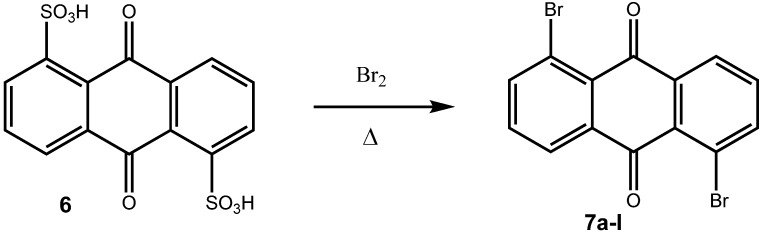
Bromination of an anthraquinone-disulfonic acid to a dibromoanthraquinone [11].

Based on our good experience in using thiophene *S*-oxides, either *in situ* [12,13,14] or in purified form [15,16,17], as dienes in the preparation of multi-functionalised arenes [18,19], we decided to utilize halogenated thiophene *S*-oxides as transient intermediates to prepare bromoanthraquinones. While most thiophenes themselves are unreactive or react sluggishly [20,21,22] and thiophene *S*,*S*-dioxides [23] often necessitate high temperatures to participate in [4+2]-cycloaddition reactions, thiophene *S*-oxides have been found to be reactive dienes in Diels-Alder type reactions. While a number of thiophene *S*-oxides [15,16,17,24,25,26,27,28], especially those with electron donating substituents have been isolated, thiophene *S*-oxides can be reacted *in situ* [12,13,14]. Thiophene *S*-oxides undergo cycloaddition reactions, when thiophenes are oxidized in the presence of a dienophile.

**Scheme 4:**
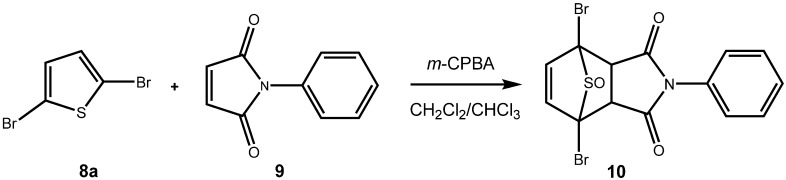
In situ preparation and cycloaddition of a dibrominated thiophene *S*-oxide [30].

From our understanding, in halogenated thiophenes, the sulfur is more difficult to oxidize with peracids or with hydrogen peroxide than in the corresponding donor substituted thiophenes. On the other hand, oxidized halothiophenes – halothiophene *S*-oxides and halothiophene *S*,*S*-dioxides – should be more reactive dienes than their electron-donor substituted counterparts. Therefore, in all likelihood, halothiophene *S*-oxides would have to be used *in situ*. In fact, Torssell has reported on one example of a successful oxidative cycloaddition of a monobrominated thiophene with 1,4-naphthoquinone (**3a**), where the cycloadduct was produced in poor yield [29]. Our own work [30] on the oxidative cycloaddition of brominated and chlorinated thiophenes (eg, **8a**) to maleimides (eg, to **9**) indicated that halothiophene *S*-oxides can be produced *in situ* and can be reacted with electron poor dienophiles (Scheme 4).

## 2. Results and Discussion

**Scheme 5:**
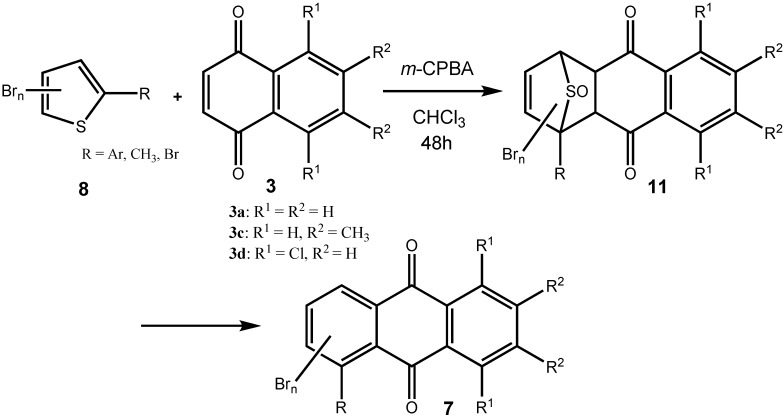
Preparation of bromoanthraquinones by oxidative cycloaddition of thiophenes to quinines.

**Figure 2:**
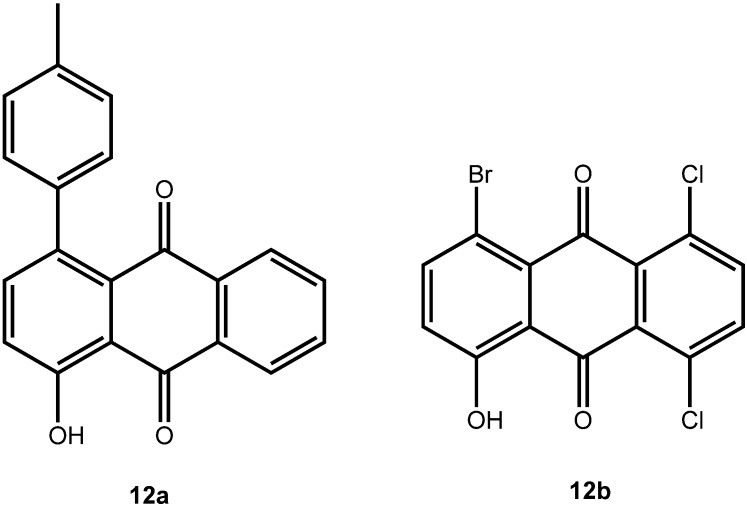
Hydroxyanthraquinones as side products in the oxidative cycloaddition reactions of thiophenes to quinines.

In the present case, a variety of brominated thiophenes **8** were submitted to oxidative cycloaddition reactions with 1,4-naphthoquinones **3**. Heated solutions of thiophene **8** and 1,4-naphthoquinone (**3a**) were treated with *meta*-chloroperbenzoic acid in small portions over 48 h. Under these conditions, cycloaddition between intermediately formed thiophene *S*-oxides and 1,4-naphthoquinone **3** takes place, where the formulated, primary sulfoxy-bridged cycloadduct **11** loses the SO-bridge with concomitant aromatization (Scheme 5). The bromoanthraquinones **7** can be obtained, albeit in very moderate yield (Table 1). A number of more polar side products formed, depending on the substrate. One important type of side product are hydroxyanthraquinones **12** (Figure 2). That bromothiophene *S*-oxides are involved here, has been shown in the reaction under analogous conditions of 2,5-dibromothiophene (**8a**), 2,3,4,5-tetrabromothiophene (**8e**) and 2,5-dichlorothiophene with *N*-phenylmaleimide (**9**), where halogenated 7-thiabicyclo[2.2.1]heptene *S*-oxides **10** could be isolated (Scheme 4) [30]. Nevertheless, even in cases where halothiophene *S*-oxides are oxidized further to halothiophene *S*,*S*-dioxides, cycloaddition reactions may be expected to proceed as electron poor thiophene *S*,*S*-dioxides have been found to undergo cycloaddition reactions readily [31,32,33], so that under the present conditions, halothiophene *S*,*S*-dioxides can also contribute to the reaction.

**Table 1:** Preparation of bromoanthraquinones by oxidative cycloaddition of thiophenes to quinines.

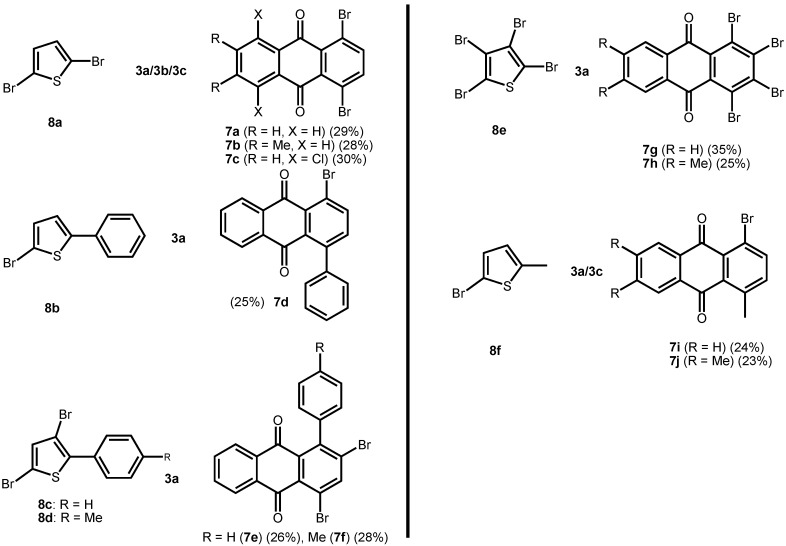

**Figure 3:**
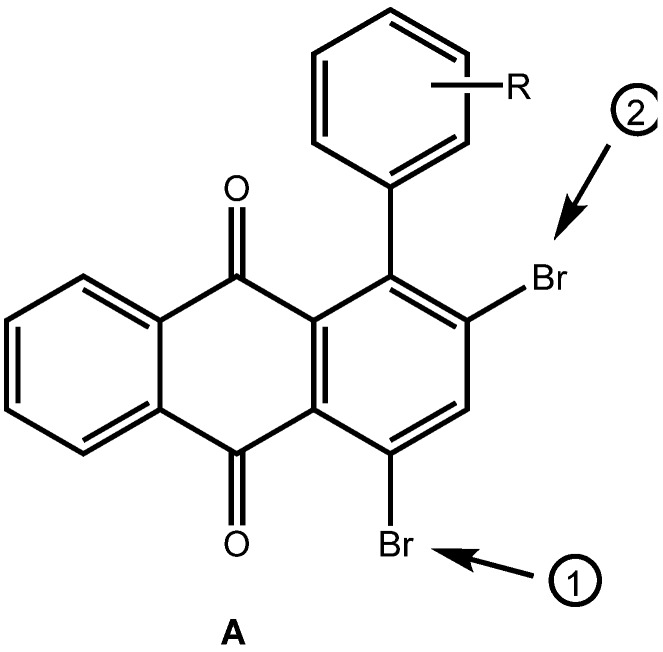
Order of entry of further aryl substituents by Suzuki-Miyaura cross-coupling reaction.

The brominated anthraquinones obtained were subjected to Suzuki-Miyaura cross coupling reactions with a variety of arylboronic acids. Either Pd(PPh_3_)_4_/PPh_3_ or Pd(PPh_3_)_2_Cl_2_/PPh_3_ was used as catalyst in a biphasic reaction medium of DME and aq. Na_2_CO_3_. The corresponding arylated anthraquinones were obtained in good yield. In the case of the 1-aryl-2,4-dibromoanthraquinones, the first aryl group enters selectively into the 4-position, *ie.*, away from the aryl function already present in the anthraquinone system (Figure 3). Prolonged reaction times and an excess of arylboronic acid make the 2-position accessible, also. In this manner it is possible to provide anthraquinones with three different aryl substituents in positions 1, 2 and 4. 

Equally interesting is the fact that chlorinated anthraquinones exchange the chloro-substituent readily, and thus they undergo Suzuki-Miyaura cross coupling reactions with ease, too, even when using a common catalyst such as Pd(PPh_3_)_4_. Thus, 1,4-dibromo-5,8-dichloroanthraquinone (**7c**) can be converted to the 1,4,5,8-tetra-arylanthraquinone **4t** (see continued Table 2), using Pd(PPh_3_)_4_ as a catalyst, as can be 1-bromo-5,8-dichloro-4-hydroxyanthraquinone (**12b**) to **13** (Scheme 7).

**Scheme 6:**
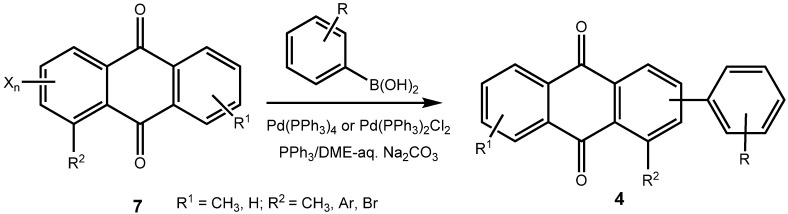
Arylated anthraquinones by Suzuki-Miyaura coupling of dibromoanthraquinones.

**Table 2:** Arylated anthraquinones by Suzuki-Miyaura coupling of dibromoanthraquinones.

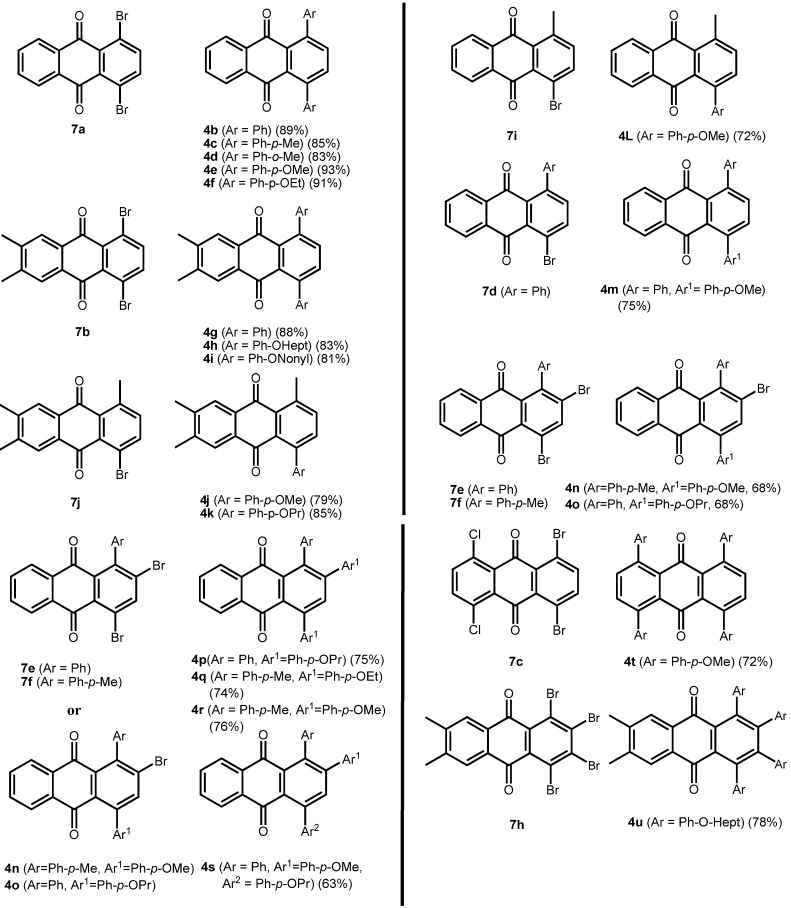

**Scheme 7:**
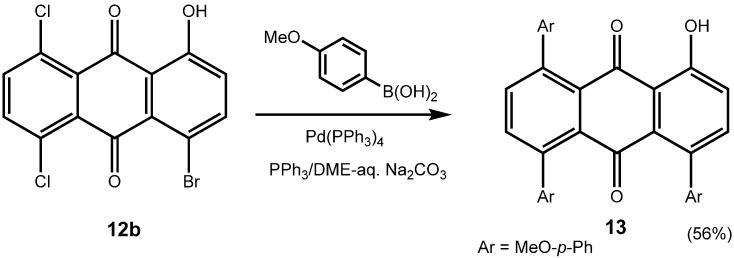
Suzuki-Miyaura coupling of chloroanthraquinones.

The anthraquinones obtained show spectral data typical for this species of compounds. Thus, in the mass spectra, many of the anthraquinones prepared above have [M^+^-CO] and [M^+^-2CO] fragmentation peaks that are typical for anthraquinones [34,35]. In their carbon NMR spectra, the carbonyl functions resonate at 184 – 185 ppm. In 1,2-aryl-substituted anthraquinones, the influence of the proximity of the *π*-system of one aryl group on the protons of the other can be noted by a high-field shift. 

The UV-VIS spectra of most of the solutions of the arylated anthraquinones in acetonitrile show at least three distinct bands, usually associated with *π*-π* transitions [36,37]. The strongest band, normally called a ‘benzoid band’ [37], is located at around *λ* = 250 nm for most of the compounds, which is in accordance to data gathered from other substituted anthraquinones. It could be shown that the substitution pattern of the aryl substituent in the anthraquinone has little influence on the wavelength of this absorption band. Methylation of the C6/C7 positions in the anthraquinone core leads to a shift of Δ*λ*= 10 nm, where *λ_max_* = 263 nm. A longer-wave *π*-π*-transition (often called a ‘quinoid band’ [37]) can be found as a shoulder at *λ* = 265 – 270 nm for the 1,4-diarylated anthraquinones. Again, there is very little influence of the substitution pattern of the aryl groups at C1 and C4 on the wavelength of this band. Also, 1,2,4-triarylated anthraquinones show this band within the same wavelength region. Where identifiable, this transition is shifted to lower energy for 6,7-methylated anthraquinones (eg., for **4k**, *λ* = 279 nm). A shift to higher wavelength is also found for the *β*-bromo substituted anthraquinone **4n **(*λ* = 275 nm). Two further *π*-π* transitions can be noted, although they cannot be identified for all compounds measured. The first is found at around *λ* = 300 nm. The *π*-π* transition with the longest wavelength can be noted at *λ* = 350 – 380 nm for the compounds measured. Substituent dependence of this transition has been reported for mono-substituted anthraquinones [36,37], and also in our case a substituent-dependence can be noted.

## 3. Experimental Section

**Warning**: *Working with meta-chloroperoxybenzoic acid at elevated temperatures is hazardous. The reactions should be carried out in a well-ventilated hood. Protections against an explosion should be set up.* (The authors themselves have not experienced any difficulties with these reactions. The above measures may be seen as protective precautions).

### General

Melting points were measured on a Yanaco microscopic hot stage and are uncorrected. IR spectra were measured with JASCO IR-700 and Nippon Denshi JIR-AQ2OM machines. ^1^H- and ^13^C-NMR spectra were recorded with a JEOL EX-270 (^1^H at 270 MHz and ^13^C at 67.8 MHz) and JEOL Lambda 400 spectrometer (^1^H at 395 MHz and ^13^C at 99.45 MHz). The chemical shifts are relative to TMS (solvent CDCl_3_, unless otherwise noted). Mass spectra were measured with a JMS-01-SG-2 spectrometer [electron impact mode (EI), 70 eV or fast atom bombardment (FAB)]. Column chromatography was carried out on Wakogel 300.

The oxidative cycloaddition reactions were carried out with commercially available *meta*-chloroperbenzoic acid (*m*-CPBA, 70-75 w%, Acros), which was used without further purification. Pd(PPh_3_)_4_ (TCI), Pd(PPh_3_)_2_Cl_2_ (TCI), 2,5-dibromothiophene (**8a**) (Aldrich), 2-methylthiophene (TCI), 2-bromothiophene (Aldrich), thiophene (Wako) and 2,5-dichlorothiophene (Aldrich) were acquired commercially. 2,3,4,5-Tetrabromothiophene (**8e**) (thiophene, Br_2_, CHCl_3_) [38], 2-bromo-5-methyl-thiophene (**8f**) (2-methylthiophene, NBS, CHCl_3_, AcOH), 2-bromo-5-phenylthiophene (**8b**) and 2-bromo-5-(*p*-tolyl)thiophene (a. 2-bromothiophene, Aryl-B(OH)_2_, Pd(PPh_3_)_4_, DME, aq. Na_2_CO_3_; b. *N*-bromosuccinimide [NBS], CHCl_3_, AcOH) [39] were prepared analogous to known procedures. 2,4-Dibromo-5-arylthiophenes, **8c** and **8d**, were synthesized by brominating 2-arylthiophenes using an excess [40] of NBS. 5,8-Dichloro-1,4-naphthoquinone (**3c**) was prepared by oxidative cycloaddition of 2,5-dichlorothiophene to *p*-benzoquinone [29]. 2,3-Dimethyl-5,8-naphthoquinone (**3b**) was prepared by cycloaddition of 2,3-dimethylbuta-1,3-diene to *p*-benzoquinone under EuCl_3_ catalysis (96 h, ClCH_2_CH_2_Cl, rt) [41] with subsequent base catalysed enolisation [42,43] of the 4a,5,8,8a-tetrahydro-6,7-dimethyl-1,4-naphthoquinone formed and oxidation of the 6,7-dimethyl-5,8-dihydronaphthalene-1,4-diol (Ag_2_O, Na_2_SO_4_, benzene) [44] to 6,7-dimethyl-5,8-dihydro-1,4-naphthoquinone, which in a last step was dehydrogenated (DDQ, benzene, reflux). *p*-Methoxyphenylboronic acid (TCI), *o*-methoxyphenylboronic acid (TCI), phenylboronic acid (TCI), and *p*-tolylboronic acid (Aldrich) were acquired commercially. *p*-Ethoxy- and *p*-propoxyphenylboronic acids were prepared from the corresponding *p*-alkoxy-bromobenzenes (a. *n*-BuLi, B(OEt)_3_, THF; b. HCl) [45].

*1,4-Dibromoanthraquinone* (**7a**) [11,46]. To a stirred solution of dibromothiophene (**8a**, 1.00 g, 4.16 mmol) and 1,4-naphthoquinone (**3a**, 517 mg, 3.47 mmol) in CHCl_3_ (20 mL) at 75 ºC was added *m*-CPBA (70w%, 4.76 g) in small portions. After 48 h, the mixture was cooled and poured into an aq. sat. Na_2_CO_3_ solution. After the mixture was stirred for 15 min. at rt, it was extracted with chloroform (3 X 25 mL). The organic phase was dried over anhydrous MgSO_4_ and concentrated *in vacuo*. The residue was subjected to column chromatography on silica gel (hexane/ether/CHCl_3_ 8:1:1) to give **7a** (370 mg, 29%); *δ*_H_ (270 MHz, CDCl_3_) 7.78 – 7.81 (2H, m), 7.81 (2H, s), 8.20 – 8.23 (2H, m); *δ*_C_ (67.8 MHz, CDCl_3_) 122.1 (2C, C_quat_), 126.9 (2C, CH), 133.5 (2C, C_quat_), 133.6 (2C, C_quat_), 134.2 (2C, CH), 140.6 (2C, CH), 181.6 (2C, C_quat_, CO); MS (EI, 70 eV) m/z (%) 368 ([^81^Br_2_]M^+^) (50), 366 ([^81^Br^79^Br]M^+^) (100), 364 ([^79^Br_2_]M^+^) (51), 340 ([^81^Br_2_]M^+^-CO) (15), 338 ([^81^Br^79^Br]M^+^-CO) (30), 336 ([^79^Br_2_]M^+^ - CO) (15), 312 ([^81^Br_2_]M^+^-2CO) (10), 310 ([^81^Br^79^Br]M^+^-2CO) (21), 308 ([^79^Br_2_]M^+^-2CO) (11), 287 (11), 285 (11), 231 (15), 229 (15), 150 (73). HRMS Found: 365.8716. Calcd. for C_14_H_6_O_2_^79^Br^81^Br: 365.8715.

### Selected data of other bromoanthraquinones

*1,4-Dibromo-6,7-dimethylanthraquinone* (**7b**). Yellow solid; *δ*_H_ (270 MHz, CDCl_3_) 2.42 (6H, s, 2 CH_3_), 7.77 (s, 2H), 7.94 (s, 2H); *δ*_C_ (67.8 MHz, CDCl_3_) 20.3 (2C, CH_3_), 122.1 (2C, C_quat_), 127.8 (2C, CH), 131.5 (2C, C_quat_), 133.7 (2C, C_quat_), 140.4 (2C, CH), 144.5 (2C, C_quat_), 181.8 (2C, C_quat_, CO); MS (EI, 70 eV) *m/z* (%) 396 ([^81^Br_2_]M^+^, 50), 394 ([^81^Br^79^Br]M^+^, 100), 392 ([^79^Br_2_]M^+^, 50), 368 ([^81^Br_2_]M^+^-CO, 12), 366 ([^81^Br^79^Br]M^+^ - CO, 25), 364 ([^79^Br_2_]M^+^ - CO, 13). HRMS Found: 393.9033. Calcd. for C_16_H_10_O_2_^79^Br^81^Br: 393.9028.

*1,4-Dibromo-5,8-dichloroanthraquinone* (**7c**). Colorless solid; *δ*_H_ (270 MHz, CDCl_3_) 7.60 (2H, s), 7.72 (2H, s); MS (EI, 70 eV) *m/z* 438 (3.3), 436 (9.2), 434 (9.6), 432 (3.9), 149 (34), 58 (100). HRMS Found: 433.7930. Calcd. for C_14_H_4_O_2_^35^Cl^37^Cl^79^Br_2_: 433.7933.

*2,4-Dibromo-1-(4-methylphenyl)anthraquinone* (**7f**). Yellow solid, mp. 183 ºC; *δ*_H_ (270 MHz, CDCl_3_) 2.47 (3H, s, CH_3_), 7.01 (2H, d, ^3^*J* = 8.1 Hz), 7.31 (2H, d, ^3^*J* = 8.1 Hz), 7.50 – 7.80 (2H, m), 7.96 – 8.00 (1H, m), 8.20 – 8.23 (1H, m), 8.38 (1H, s); *δ*_C_ (67.8 MHz, CDCl_3_) 21.6, 122.1, 126.9, 127.6 (2C), 128.0, 129.0, 129.2 (2C), 131.3, 133.2, 133.4, 133.5, 134.0, 134.1, 137.3, 137.4, 143.6, 143.9, 182.1, 182.2; MS (EI, 70 eV) *m/z* (%) 456 ([^81^Br^79^Br]M^+^) (18), 299 (100). HRMS Found: 455.9188. Calcd. for C_21_H_12_O_2_^81^Br^79^Br: 455.9185.

*1,2,3,4-Tetrabromoanthraquinone* (**7g**) [47]. Orange solid; mp. 200 ºC; *δ*_H_ (270 MHz, CDCl_3_) 7.76 – 7.79 (2H, m), 8.11 – 8.14 (2H, m); *δ*_C_ (67.8 MHz, CDCl_3_) 125.0 (2C, C_quat_), 126.8 (2C, CH), 133.6 (2C, C_quat_), 134.3 (2C, CH), 139.0 (2C, C_quat_), 181.8 (2C, C_quat_, CO); MS (FAB, 3-nitrobenzyl alcohol) *m/z* (%) 527 ([^81^Br_3_^79^Br]MH^+^) (0.2), 526 ([^81^Br_3_^79^Br]M^+^) (0.1), 525 ([^81^Br_2_^79^Br_2_]MH^+^) (0.3), 524 ([^81^Br_2_^79^Br_2_]M^+^) (0.2), 523 ([^81^Br^79^Br_3_]MH^+^) (0.2). HRMS Found: 524.6993. Calcd. for C_14_H_5_O_2_^79^Br_2_^81^Br_2_: 524.6983 (MH^+^, FAB).

*1,2,3,4-Tetrabromo-6,7-dimethylanthraquinone* (**7h**). Slowly solidifying yellow oil; *δ*_H_ (270 MHz, CDCl_3_) 2.42 (6H, s, 2 CH_3_), 7.86 (2H, s); *δ*_C_ (67.8 MHz, CDCl_3_) 20.3 (2C, CH_3_), 127.7 (2C), 131.5 (2C), 134.5 (2C), 135.4 (2C), 138.7 (2C), 144.5 (2C), 181.8 (2C, CO); MS (FAB, 3-nitrobenzyl alcohol) *m/z* (%) 552 ([^81^Br_2_^79^Br_2_]M^+^) (0.2)

*1-Bromo-4-methylanthraquinone* (**7i**) [48]. Beige colored solid; *δ*_H_ (270 MHz, CDCl_3_) 2.79 (3H, s, CH_3_), 7.36 (1H, d, ^3^*J* = 8.4 Hz), 7.74 – 7.78 (2H, m), 7.87 (1H, d, ^3^*J* = 8.4 Hz), 8.15 – 8.24 (2H, m); *δ*_C_ (67.8 MHz, CDCl_3_) 23.6 (CH_3_), 120.2 (C_quat_), 126.6 (CH), 126.9 (CH), 127.8 (C_quat_), 132.9 (C_quat_), 133.8 (2C, CH), 134.4 (C_quat_), 137.9 (CH), 140.2 (CH), 140.4 (C_quat_), 141.9 (C_quat_), 182.9 (C_quat_, CO), 184.5 (C_quat_, CO); MS (EI, 70 eV) *m/z* (%) 302 ([^81^Br]M^+^, 97), 300 ([^79^Br]M^+^, 100), 274 ([^81^Br]M^+^-CO, 15), 272 ([^79^Br]M^+^-CO, 15), 193 (59), 165 (90). HRMS Found: 301.9764. Calcd. for C_15_H_9_O_2_^81^Br: 301.9767. Found: 299.9789. Calcd. for C_15_H_9_O_2_^79^Br: 299.9786.

*1-Bromo-4,6,7-trimethylanthraquinone* (**7j**). Yellow solid, mp. 194 ºC; *δ*_H_ (270 MHz, CDCl_3_) 2.42 (6H, s, 2 CH_3_), 2.73 (3H, s, CH_3_), 7.33 (1H, d,^ 3^*J* = 8.4 Hz), 7.82 (1H, d, ^3^*J* = 8.4 Hz), 7.91 (1H, s), 7.95 (1H, s); *δ*_C_ (67.8 MHz, CDCl_3_) 20.2 (2C, CH_3_), 20.7 (CH_3_), 120.0 (C_quat_), 127.4 (CH), 127.8 (CH), 131.7 (C_quat_), 131.8 (C_quat_), 132.6 (C_quat_), 134.1 (C_quat_), 137.7 (CH), 140.0 (CH), 141.8 (C_quat_), 143.8 (C_quat_, 2C), 183.2 (C_quat_, CO), 184.8 (C_quat_, CO); MS (EI, 70 eV) *m/z* (%) 330 ([^81^Br]M^+^) (100), 328 ([^79^Br]M^+^) (100), 315 ([^81^Br]M^+^-CH_3_) (38), 313 ([^79^Br]M^+^-CH_3_) (39), 302 ([^81^Br]M^+^-CO) (26), 300 ([^81^Br]M^+^-CO) (28), 287 ([^81^Br]M^+^-CH_3_-CO) (26), 285 ([^79^Br]M^+^-CO-CH_3_) (26), 221 (55), 178 (83). HRMS Found: 328.0097. Calcd. for C_17_H_13_O_2_^79^Br: 328.0099.

*1,4-Bis(4-methylphenyl)anthraquinone* (**4c**) [49]. Under an inert atmosphere, a solution of **7a** (324 mg, 0.89 mmol), 4-methylphenylboronic acid (385 mg, 2.83 mmol), Pd(PPh_3_)_2_Cl_2_ (30 mg, 4.0^.^10^-5^ mol) and triphenylphosphine (30 mg, 0.11 mmol) in a solvent mixture of DME (10 mL) and aq. Na_2_CO_3_ (2.32 g Na_2_CO_3_ in 15 mL H_2_O, 6 mL) was kept at 65 ºC for 18h. Thereafter the cooled solution was poured into water (25 mL) and extracted with chloroform (3 x 15 mL). The combined organic phase was dried over anhydrous MgSO_4_ and was concentrated *in vacuo*. Column chromatography of the residue on silica gel (hexane/CHCl_3_/ether 3:1:1) gave **4c** (293 mg, 85%) as an orange solid; mp. 265 ºC; *δ*_H_ (270 MHz, CDCl_3_) 2.45 (6H, s, 2 CH_3_), 7.18 (4H, d, ^3^*J* = 7.6 Hz), 7.27 (4H, d, ^3^*J* = 7.6 Hz), 7.53 (2H, s), 7.65 – 7.70 (2H, m), 8.05 - 8.09 (2H, m); *δ*_C_ (67.8 MHz, CDCl_3_) 21.3 (2C, CH_3_), 126.7 (2C, CH), 127.9 (4C, CH), 128.9 (4C, CH), 132.8 (2C, C_quat_), 133.7 (2C, CH), 134.1 (2C, C_quat_), 136.5 (2C, CH), 136.8 (2C, C_quat_), 139.4 (2C, C_quat_), 143.9 (2C, C_quat_), 184.2 (2C, CO); MS (EI, 70 eV) *m/z* (%) = 388 (M^+^) (83), 373 (M^+^-CH_3_) (100), 179 (40). HRMS Found: 388.1469. Calcd. for C_28_H_20_O_2_: 388.1463. Found: C, 84.36; H, 5.12%. Calcd. for C_28_H_20_O_2_^.^H_2_O: C, 84.61; H, 5.33%. UV-Vis spectrum (CH_3_CN, nm) *λ*_max_ 253 (44700), 268 (sh, 21310), 298 (9350), 358 (2470).

### Selected data for other arylated anthraquinones

*1,4-Diphenylanthraquinone* (**4b**) [2,50] Yellow solid; *δ*_H_ (270 MHz, CDCl_3_) 7.29 – 7.35 (4H, m), 7.43 – 7.48 (6H, m), 7.56 (2H, s), 7.66 – 7.71 (2H, m), 8.05 – 8.08 (2H, m); *δ*_C_ (67.8 MHz, CDCl_3_) 126.8 (2C, CH), 127.2 (2C, CH), 127.9 (4C, CH), 128.2 (4C, CH), 132.7 (2C, C_quat_), 133.7 (2C, CH), 134.0 (2C, C_quat_), 136.4 (2C, CH), 142.3 (2C, C_quat_), 144.1 (2C, C_quat_), 184.0 (2C, C_quat_, CO); MS (FAB, 3-nitrobenzyl alcohol) *m/z* (%) 361 (MH^+^) (5.6). HRMS Found: 361.1232. Calcd. for C_26_H_17_O_2_: 361.1229 (MH^+^, FAB); UV-Vis spectrum (CH_3_CN, nm) *λ*_max_ 253 (36370), 269 (sh, 19190), 288 (sh, 7320).

*1,4-Bis(4-methoxyphenyl)anthraquinone* (**4e**). Orange needles; mp. 231 ºC; *δ*_H_ (270 MHz, CDCl_3_) 3.89 (6H, s, 2 OCH_3_), 7.00 (4H, d, ^3^*J* = 8.6 Hz), 7.26 (4H, d, ^3^*J* = 8.6 Hz), 7.53 (2H, s), 7.68 – 7.72 (2H, m), 8.06 – 8.09 (2H, m); *δ*_C_ (67.8 MHz, CDCl_3_) 55.2 (2C, OCH_3_), 113.7 (4C, CH), 126.7 (2C, CH), 129.3 (4C, CH), 133.7 (2C, CH), 132.9 (2C, C_quat_), 134.1 (2C, C_quat_), 134.5 (2C, C_quat_), 136.6 (2C, CH), 143.6 (2C, C_quat_), 158.9 (2C, C_quat_), 184.3 (2C, C_quat_, CO); MS (EI, 70 eV) *m/z* (%) 420 (M^+^) (100), 389 (32), 333 (18), 313 (13), 276 (17). HRMS Found: 420.1367. Calcd. for C_28_H_20_O_4_: 420.1362; UV-Vis spectrum (CH_3_CN, nm) *λ*_max_ 253 (59610), 271 (sh, 23890), 313 (13280).

*1,4-Bis(4-ethoxyphenyl)anthraquinone* (**4f**). Orange needles; mp. 239 ºC; *δ*_H_ (270 MHz, CDCl_3_) 1.47 (3H, t, CH_3_, ^3^*J* = 7.0 Hz), 4.12 (2H, q, OCH_2_,^ 3^*J* = 7.0 Hz), 6.98 (4H, d, ^3^*J* = 8.4 Hz), 7.24 (4H, d, ^3^*J* = 8.4 Hz), 7.53 (2H, s), 7.67 – 7.70 (2H, m), 8.05 – 8.09 (2H, m); *δ*_C_ (67.8 MHz, CDCl_3_) 14.9 (2C, CH_3_), 63.4 (2C, OCH_2_), 114.2 (4C, CH), 126.7 (2C, CH), 129.3 (4C, CH), 132.9 (2C, C_quat_), 133.6 (2C, CH), 134.2 (2C, C_quat_), 134.3 (2C, C_quat_), 136.6 (2C, CH), 143.6 (2C, C_quat_), 158.3 (2C, C_quat_), 184.3 (2C, C_quat_, CO); MS (FAB, 3-nitrobenzyl alcohol) *m/z* (%) 449 (MH^+^) (7.5). HRMS Found: 449.1749. Calcd. for C_30_H_25_O_4_: 449.1753. Found: C, 79.57; H, 5.47%. Calcd. for C_30_H_24_O_4_^.^0.2H_2_O: C, 79.70; H, 5.44%; UV-Vis spectrum (CH_3_CN, nm) *λ*_max_ 253 (49430), 269 (sh, 21220), 314 (10910).

*1,4-Diphenyl-6,7-dimethylanthraquinone* (**4g**). Yellow needles, mp. 232 ºC; *δ*_H_ (270 MHz, CDCl_3_) 2.34 (6H, s, 2 CH_3_), 7.30 – 7.34 (4H, m), 7.43 – 7.50 (6H, m), 7.53 (2H, s), 7.82 (2H, s); *δ*_C_ (67.8 MHz, CDCl_3_) 20.1 (2C, CH_3_), 127.0 (2C, CH), 127.7 (2C, CH), 127.9 (4C, CH), 128.1 (4C, CH), 132.0 (2C, C_quat_), 132.9 (2C, C_quat_), 136.1 (2C, CH), 142.5 (2C, C_quat_), 143.7 (2C, C_quat_), 143.9 (2C, C_quat_), 184.1 (2C, C_quat_, CO); MS (FAB, 3-nitrobenzyl alcohol) *m/z* (%) 389 (MH^+^) (5.3). HRMS Found: 389.1539. Calcd. for C_28_H_21_O_2_: 389.1542 (FAB). Found: C, 85.80; H, 5.18%. Calcd. for C_28_H_20_O_2_^.^0.2H_2_O: C, 85.78; H, 5.24%; UV-Vis spectrum (CH_3_CN, nm) *λ*_max_ 263 (41490), 338 (4480).

*1,4-Bis(4-heptoxyphenyl)-6,7-dimethylanthraquinone* (**4h**). Beige solid; mp. 181 ºC; *δ*_H_ (270 MHz, CDCl_3_) 0.92 (6H, t, ^3^*J* = 6.2 Hz, 2 CH_3_), 1.34 – 1.60 (16H, m), 1.78 – 1.86 (4H, m), 2.35 (6H, s, 2 CH_3_), 4.03 (4H, t, ^3^*J* = 6.5 Hz), 6.98 (4H, d, ^3^*J* = 8.6 Hz), 7.23 (4H, d, ^3^*J* = 8.6 Hz), 7.50 (2H, s), 7.83 (2H, s); *δ*_C_ (67.8 MHz, CDCl_3_) 14.1 (2C, CH_3_), 20.2 (2C, CH_2_), 22.7 (2C, CH_2_), 26.1 (2C, CH_2_), 29.1 (2C, CH_2_), 29.4 (2C, CH_2_), 31.8 (2C, CH_2_), 67.9 (2C, OCH_2_), 114.1 (4C, CH), 127.7 (2C, CH), 129.2 (4C, CH), 132.1 (2C, C_quat_), 133.0 (2C, C_quat_), 134.4 (2C, C_quat_), 136.4 (2C, C_quat_), 143.5 (2C, C_quat_), 158.4 (2C, C_quat_), 184.5 (2C, C_quat_, CO); MS (FAB, 3-nitrobenzyl alcohol) *m/z* (%) 617 (MH^+^) (0.5). HRMS Found: 617.3629. Calcd. for C_42_H_49_O_4_: 617.3631 (MH^+^, FAB)

*1,4-Bis(4-nonyloxyphenyl)-6,7-dimethylanthraquinone* (**4i**). Yellow-orange solid; mp. 176 ºC; *δ*_H_ (270 MHz, CDCl_3_) 0.88 (6H, t, ^3^*J* = 4.3 Hz, 2 CH_3_), 1.30 (20H, m), 1.44 – 1.49 (4H, m), 1.78 – 1.85 (4H, m), 2.35 (6H, s, 2 CH_3_), 4.03 (4H, t, ^3^*J* = 6.5 Hz), 6.98 (4H, d, ^3^*J* = 8.6 Hz), 7.23 (4H, d, ^3^*J* = 8.6 Hz), 7.50 (2H, s), 7.83 (2H, s); *δ*_C_ (67.8 MHz, CDCl_3_) 14.1 (2C, CH_3_), 20.2 (2C, CH_2_), 22.7 (2C, CH_2_), 26.1 (2C, CH_2_), 29.3 (2C, CH_2_), 29.4 (2C, CH_2_), 29.5 (2C, CH_2_), 29.6 (2C, CH_2_), 31.9 (2C, CH_2_), 67.9 (2C, OCH_2_), 114.1 (4C, CH), 127.6 (2C, CH), 129.2 (4C, CH), 132.1 (2C, C_quat_), 133.0 (2C, C_quat_), 134.4 (2C, C_quat_), 136.4 (2C, C_quat_), 143.5 (2C, C_quat_), 158.4 (2C, C_quat_), 184.5 (2C, C_quat_, CO); MS (FAB, 3-nitrobenzyl alcohol) *m/z* (%) 673 (MH^+^) (100). HRMS Found: 673.4254. Calcd. for C_46_H_57_O_4_: 673.4257 (MH_+_, FAB).

*1-(4-Methoxyphenyl)-4,6,7-trimethylanthraquinone* (**4j**). Solid; *δ*_H_ (270 MHz, CDCl_3_) 2.30 (3H, s, CH_3_), 2.40 (3H, s, CH_3_), 2.86 (3H, s, CH_3_), 3.87 (3H, s, OCH_3_), 6.96 (2H, d, ^3^*J* = 8.6 Hz), 7.19 (2H, d, ^3^*J* = 8.6 Hz), 7.40 (1H, d, ^3^*J* = 7.8 Hz), 7.50 (1H, d, ^3^*J* = 7.8 Hz), 7.80 (1H, s), 7.94 (1H, s); *δ*_C_ (67.8 MHz, CDCl_3_) 20.1 (CH_3_), 20.2 (CH_3_), 23.8 (CH_3_), 55.2 (OCH_3_), 113.5 (2C, CH), 127.4 (CH), 127.5 (CH), 129.1 (2C, CH), 132.1 (C_quat_), 132.2 (C_quat_), 132.9 (C_quat_), 133.0 (C_quat_), 135.1 (C_quat_), 136.7 (CH), 136.8 (CH), 141.1 (C_quat_), 142.5 (C_quat_), 143.3 (C_quat_), 143.4 (C_quat_), 158.6 (C_quat_), 184.7 (C_quat_, CO), 185.9 (C_quat_, CO); MS (EI, 70 eV) m/z (%) 356 (M^+^) (84), 355 (100), 325 (32), 312 (14). HRMS Found: 356.1413. Calcd. for C_24_H_20_O_3_: 356.1412; UV-Vis spectrum (CH_3_CN, nm) *λ*_max_ 263 (49630), 278 (sh, 19470), 339 (4880).

*1-(4-Propoxyphenyl)-4,6,7-trimethylanthraquinone* (**4k**). Yellow solid; mp. 215 ºC; *δ*_H_ (270 MHz, CDCl_3_) 1.07 (3H, t, ^3^*J* = 7.6 Hz, CH_3_), 1.56 (3H, s, CH_3_), 1.85 (2H, dt, ^3^*J* = 7.6 Hz, ^3^*J* = 6.5 Hz), 2.35 (3H, s, CH_3_), 2.41 (3H, s, CH_3_), 3.99 (2H, t, ^3^*J* = 6.5 Hz, OCH_2_), 6.95 (2H, d, ^3^*J* = 8.6 Hz), 7.17 (2H, d, ^3^*J* = 8.6 Hz), 7.41 (1H, d, ^3^*J* = 7.8 Hz), 7.50 (1H, d, ^3^*J* = 7.8 Hz), 7.81 (1H, s), 7.94 (1H, s); *δ*_C_ (67.8 MHz, CDCl_3_) 10.6 (CH_3_), 20.1 (CH_3_), 20.2 (CH_3_), 22.7 (CH_2_), 23.8 (CH_3_), 69.4 (OCH_2_), 114.1 (2C, CH), 127.5 (CH), 127.6 (CH), 129.1 (2C, CH), 132.1 (C_quat_), 132.2 (C_quat_), 132.8 (C_quat_), 133.0 (C_quat_), 134.8 (C_quat_), 136.7 (CH), 136.8 (CH), 141.1 (C_quat_), 142.6 (C_quat_), 143.3 (C_quat_), 143.4 (C_quat_), 158.2 (C_quat_), 184.7 (C_quat_, CO), 185.9 (C_quat_, CO); MS (EI, 70 eV) m/z (%) 384 (M^+^) (68), 341 (M^+^-(CH_2_)_2_CH_3_) (100). HRMs Found: 384.1718. Calcd. for C_26_H_24_O_3_: 384.1725 UV-Vis spectrum (CH_3_CN, nm) *λ*_max_ 263 (57850), 279 (sh, 20490), 330 (5580, ill-defined).

*1-(4-Methoxyphenyl)-4-methylanthraquinone* (**4l**) [50]. Yellow-orange needles; mp. 221 ºC; *δ*_H_ (270 MHz, CDCl_3_) 2.88 (3H, s, CH_3_), 3.88 (3H, s, OCH_3_), 6.97 (2H, d, ^3^*J* = 8.6 Hz), 7.20 (2H, d, ^3^*J* = 8.6 Hz), 7.44 (1H, d, ^3^*J* = 8.1 Hz), 7.53 (1H, d, ^3^*J* = 8.1 Hz), 7.67 – 7.75 (2H, m), 8.04 – 8.07 (1H, m), 8.19 – 8.23 (1H, m); *δ*_C_ (67.8 MHz, CDCl_3_) 23.8 (CH_3_), 55.2 (OCH_3_), 113.6 (2C, CH), 126.6 (CH), 129.2 (2C, CH), 132.8 (C_quat_), 132.9 (C_quat_), 133.5 (CH), 133.6 (CH), 134.1 (C_quat_), 134.2 (C_quat_), 134.8 (C_quat_), 137.0 (CH, 3C), 141.3 (C_quat_), 142.6 (C_quat_), 158.7 (C_quat_), 184.6 (C_quat_, CO), 184.7 (C_quat_, CO); MS (FAB, 3-nitrobenzyl alcohol) *m/z* (%) 329 (MH^+^) (14). HRMS Found: 329.1183. Calcd. for C_22_H_17_O_3_: 329.1178 (MH^+^, FAB). Found: C, 79.89; H, 4.73%. Calcd. for C_22_H_16_O_3_^.^0.1H_2_O: C, 80.03; H, 4.91%; UV-Vis spectrum (CH_3_CN, nm) *λ*_max_ 253 (38343), 269 (sh, 15440), 302 (5400), 354 (2505).

*1-(4-Methoxyphenyl)-4-phenylanthraquinone* (**4m**). Beige solid; MS (FAB, 3-nitrobenzyl alcohol) *m/z* (%) 391 (MH^+^) (7.6). HRMS Found: 391.1340. Calcd. for C_27_H_19_O_3_: 391.1334 (MH^+^, FAB); UV-Vis spectrum (CH_3_CN, nm) *λ*_max_ 253 (38520), 271 (sh, 18390), 306 (7980).

*2-Bromo-1-(4-methylphenyl)-4-(4-methoxyphenyl)anthraquinone* (**4n**). Orange needles; mp. 208 ºC; *δ*_H_ (270 MHz, CDCl_3_) 2.49 (3H, s, CH_3_), 3.89 (3H, s, OCH_3_), 7.01 (2H, d, ^3^*J* = 8.9 Hz), 7.08 (2H, d, ^3^*J* = 8.1 Hz), 7.27 (2H, d, ^3^*J* = 8.9 Hz), 7.33 (2H, d, ^3^*J* = 8.1 Hz), 7.66 – 7.70 (2H, m), 7.92 (1H, s), 8.00 – 8.07 (2H, m); MS (FAB, 3-nitrobenzyl alcohol) *m/z* (%) 485 ([^81^BrM]H^+^) (7.2), 484 (^81^BrM^+^) (8.0), 483 ([^79^BrM]H^+^, 8.9), 482 (^79^BrM^+^) (6.0). HRMS Found: 483.0595. Calcd. for C_28_H_20_O_3_^79^Br (MH^+^, FAB); UV-Vis spectrum (CH_3_CN, nm) *λ*_max_ 258 (37150), 275 (sh, 16870), 309 (8100).

*2-Bromo-1-phenyl-4-(4-propoxyphenyl)anthraquinone* (**4o**). Yellow solid; mp. 183 ºC; *δ*_H_ (270 MHz, CDCl_3_) 1.08 (3H, t, ^3^*J* = 7.3 Hz, CH_3_), 1.86 (2H, dt, ^3^*J* = 7.3 Hz, ^3^*J* = 6.5 Hz), 4.01 (2H, t, ^3^*J* = 6.5 Hz, OCH_2_), 7.00 (2H, d, ^3^*J* = 8.4 Hz), 7.17 – 7.21 (2H, m), 7.25 (2H, d, ^3^*J* = 8.4 Hz), 7.48 – 7.54 (3H, m), 7.66 – 7.70 (2H, m), 7.93 (1H, s), 7.98 – 8.02 (1H, m), 8.04 – 8.07 (1H, m); *δ*_C_ (67.8 MHz, CDCl_3_) 10.6 (CH_3_), 22.7 (CH_2_), 69.5 (OCH_2_), 114.3 (2C, CH), 126.7 (CH), 126.9 (CH), 127.4 (CH), 127.9 (2C, CH), 128.3 (2C, CH), 129.2 (2C, CH), 131.7 (C_quat_), 132.3 (C_quat_), 132.9 (C_quat_), 133.6 (C_quat_), 133.8 (CH), 133.9 (CH), 134.3 (C_quat_), 141.0 (CH), 141.2 (C_quat_), 142.9 (C_quat_), 145.1 (C_quat_), 155.8 (C_quat_), 183.1 (C_quat_, CO), 183.7 (C_quat_, CO); MS (EI, 70 eV) *m/z* (%) 498 ([^81^Br]M^+^) (100), 496 ([^79^Br]M^+^) (98), 455 ([^81^Br]M^+^-(CH_2_)_2_CH_3_) (84), 453 ([^79^Br]M^+^-(CH_2_)_2_CH_3_) (81). HRMS Found: 496.0677. Calcd. for C_29_H_21_O_3_^79^Br: 496.0674.

*1-(4-Methylphenyl)-2,4-bis(4-ethoxyphenyl)anthraquinone* (**4q**). Yellow needles, mp. 230 ºC; *δ*_H_ (270 MHz, CDCl_3_) 1.37 (3H, t, ^3^*J* = 7.0 Hz, CH_3_), 1.46 (3H, t, ^3^*J* = 7.0 Hz, CH_3_), 2.36 (3H, s, CH_3_), 3.97 (2H, d, ^3^*J* = 7.0 Hz, OCH_2_), 4.11 (2H, d, ^3^*J* = 7.0 Hz, OCH_2_), 6.67 (2H, d, ^3^*J* = 8.9 Hz), 6.91 – 6.99 (6H, m), 7.08 (2H, d, ^3^*J* = 7.8 Hz), 7.29 (2H, d, ^3^*J* = 8.9 Hz), 7.57 (1H, s), 8.00 – 8.04 (2H, m), 8.06 – 8.09 (2H, m); *δ*_C_ (67.8 MHz, CDCl_3_) 14.7 (CH_3_), 14.9 (CH_3_), 21.4 (CH_3_), 63.3 (OCH_2_), 63.4 (OCH_2_), 113.7 (2C, CH), 114.1 (2C, CH), 126.5 (CH), 126.7 (CH), 128.6 (2C, CH), 129.2 (2C, CH), 129.4 (2C, CH), 130.7 (2C, CH), 131.3 (C_quat_), 132.0 (C_quat_), 133.4 (CH), 133.5 (CH), 134.1 (C_quat_), 134.2 (C_quat_), 134.3 (C_quat_), 134.5 (C_quat_), 135.9 (C_quat_), 137.0 (C_quat_), 138.9 (CH), 141.6 (C_quat_), 143.4 (C_quat_), 147.4 (C_quat_), 158.0 (C_quat_), 158.3 (C_quat_), 184.1 (C_quat_, CO), 184.9 (C_quat_, CO); MS (FAB, 3-nitrobenzyl alcohol) m/z (%) 539 (MH^+^) (31). HRMS Found: 539.2219. Calcd. for C_37_H_31_O_4_: 539.2222. Found: C, 82.26; H, 5.62%. Calcd. for C_37_H_30_O_4_: C, 82.50; H, 5.61%; UV-Vis spectrum (CH_3_CN, nm) *λ*_max_ 250 (57030), 268 (sh, 31850), 298 (sh, 19440), 359 (6340).

*1-(4-Methylphenyl)-2,4-bis(4-methoxyphenyl)anthraquinone* (**4r**). Orange solid; mp. 230 ºC; *δ*_H_ (270 MHz, CDCl_3_) 2.36 (3H, s, CH_3_), 3.75 (3H, s, OCH_3_), 3.88 (3H, s, OCH_3_), 6.69 (2H, d, ^3^*J* = 8.6 Hz), 6.93 – 6.96 (4H, m), 6.99 (2H, d, ^3^*J* = 8.6 Hz), 7.08 (2H, d, ^3^*J* = 8.6 Hz), 7.31 (2H, d, ^3^*J* = 8.6 Hz), 7.57 (1H, s), 7.65 – 7.69 (2H, m), 8.00 – 8.11 (2H, m); *δ*_C_ (67.8 MHz, CDCl_3_) 21.4 (CH_3_), 55.1 (OCH_3_), 55.2 (OCH_3_), 113.1 (2C, CH), 113.6 (2C, CH), 126.5 (CH), 126.7 (CH), 128.6 (2C, CH), 129.2 (2C, CH), 129.4 (2C, CH), 130.7 (2C, CH), 131.4 (C_quat_), 132.2 (C_quat_), 133.5 (CH), 133.6 (CH), 134.1 (C_quat_), 134.3 (C_quat_), 134.5 (C_quat_), 134.6 (C_quat_), 135.9 (C_quat_), 137.0 (C_quat_), 138.9 (CH), 141.6 (C_quat_), 143.4 (C_quat_), 147.4(C_quat_), 158.6 (C_quat_), 158.9 (C_quat_), 184.1 (C_quat_, CO), 184.9 (C_quat_, CO). MS (FAB, 3-nitrobenzyl alcohol) *m/z* (%) 511 (MH^+^) (13). HRMS Found: 511.1905. Calcd. for C_35_H_27_O_4_: 511.1909 (MH^+^, FAB); UV-Vis spectrum (CH_3_CN, nm) *λ*_max_ 250 (50 085), 269 (sh, 26790), 300 (sh, 15980), 365 (4960).

*2-(4-Methoxyphenyl)-1-phenyl-4-(4-propoxyphenyl)anthraquinone* (**4s**). Light yellow needles; mp. 233 ºC; *δ*_H_ (270 MHz, CDCl_3_) 1.08 (3H, t, ^3^*J* = 7.6 Hz, CH_3_), 1.86 (2H, tt, ^3^*J* = 7.6 Hz, ^3^*J* = 6.2 Hz), 3.74 (3H, s, OCH_3_), 4.00 (t, 2H, ^3^*J* = 6.2 Hz, OCH_2_), 6.68 (2H, d, ^3^*J* = 8.6 Hz), 6.94 (2H, d, ^3^*J* = 8.6 Hz), 6.99 (2H, d,^ 3^*J* = 8.6 Hz), 7.27 – 7.30 (3H, m), 7.04 – 7.08 (2H, m), 7.30 (2H, d, ^3^*J* = 8.6 Hz), 7.59 (1H, s), 7.65 – 7.71 (2H, m), 8.00 – 8.03 (1H, m), 8.07 – 8.10 (1H, m); *δ*_C_ (67.8 MHz, CDCl_3_) 10.6 (CH_3_), 22.7 (CH_2_), 55.1 (OCH_3_), 69.4 (OCH_2_), 113.2 (CH, 2C), 114.2 (CH, 2C), 126.5 (CH), 126.6 (CH), 126.7 (CH), 127.7 (CH, 2C), 129.4 (CH, 4C), 130.7 (CH, 2C), 131.4 (C_quat_), 132.6 (C_quat_), 133.5 (CH), 133.6 (CH), 134.1 (C_quat_), 134.2 (C_quat_), 134.4 (C_quat_), 138.9 (C_quat_), 140.2 (C_quat_), 141.5 (C_quat_), 143.7 (C_quat_), 147.3 (C_quat_), 158.6 (C_quat_), 158.7 (C_quat_), 184.1 (C_quat_, CO), 184.8 (C_quat_, CO). MS (FAB, 3-nitrobenzyl alcohol) *m/z* (%) 525 (MH^+^) (9), 524 (M^+^) (11). HRMS Found: 524.1990. Calcd. For C_36_H_28_O_4_: 524.1988. UV-Vis spectrum (CH_3_CN, nm) *λ*_max_ 249 (22370), 268 (sh, 12560), 298 (sh, 7490), 382 (2320).

*1,4,5,8-Tetrakis(4-methoxyphenyl)anthraquinone* (**4t**). Pale orange solid; mp. 251 ˚C; *δ*_H_ (270 MHz, CDCl_3_) 3.84 (12H, s, 4 OCH_3_), 6.85 (8H, d, ^3^*J* = 8.4 Hz), 7.21 (8H, d, ^3^*J* = 8.4 Hz), 7.48 (4H, s); *δ*_C_ (67.8 MHz, CDCl_3_) 55.2 (4C, OCH_3_), 113.4 (8C, CH), 130.3 (8C, CH), 131.9 (4C, C_quat_), 134.5 (4C, CH), 135.5 (4C, C_qua_t), 140.3 (4C, C_quat_), 159.0 (4C, C_quat_), 188.4 (2C, C_quat_, CO); MS (FAB, 3-nitrobenzyl alcohol) *m/z* (%) 633 (MH^+^) (1.0). HRMS Found: 633.2286. Calcd. for C_42_H_33_O_6_: 633.2277 (MH^+^, FAB). 

*1,2,3,4-Tetrakis(4-heptoxyphenyl)anthraquinone* (**4u**). Slowly crystallizing orange oil; *δ*_H_ (270 MHz, CDCl_3_) 0.88 (12H, t, ^3^*J* = 7.0 Hz, 4 CH_3_), 1.28 – 1.31 (32H, m), 1.66 (4H, m), 1.72 (4H, m), 2.32 (6H, s, 2 CH_3_), 3.74 (4H, t, ^3^*J* = 6.5 Hz), 3.90 (4H, t, ^3^*J* = 6.5 Hz), 6.39 (4H, d, ^3^*J* = 8.6 Hz), 6.52 (4H, d, ^3^*J* = 8.6 Hz), 6.70 (4H, d, ^3^*J* = 8.6 Hz), 6.86 (4H, d, ^3^*J* = 8.6 Hz), 7.78 (2H, s); *δ*_C_ (67.8 MHz, CDCl_3_) 14.0 (2C, CH_3_), 14.1 (2C, CH_3_), 20.1 (2C), 22.5 (2C), 22.6 (2C), 25.9 (2C), 26.1 (2C), 29.0 (2C), 29.1 (2C), 29.2 (2C), 29.4 (2C), 31.7 (2C), 31.8 (2C), 67.7 (4C, OCH_2_), 112.9 (4C), 113.5 (4C), 128.5 (2C), 130.2 (4C), 131.2 (2C), 131.6 (4C), 132.4 (2C), 132.8 (2C), 133.2 (2C), 142.6 (2C), 143.2 (2C), 148.3 (2C), 157.2 (2C), 156.7 (2C), 184.8 (2C, CO); MS (FAB, 3-nitrobenzyl alcohol) *m/z* (%) 997 (MH^+^) (100). HRMS Found: 997.6355. Calcd. for C_68_H_85_O_5_: 997.6346.

*1-Hydroxy-4,5,8-tris(4-methoxyphenyl)anthraquinone* (**13**). Reddish solid; mp. 238 ˚C; *δ*_H_ (270 MHz, CDCl_3_) 3.82 (3H, s, OCH_3_), 3.83 (3H, s, OCH_3_), 3.90 (3H, s, OCH_3_), 6.84 (2H, d, ^3^*J* = 8.6 Hz), 6.87 (2H, d, ^3^*J* = 8.6 Hz), 7.00 (2H, d, ^3^*J* = 8.6 Hz), 7.14 – 7.29 (7H, m), 7.46 (1H, d, ^3^*J* = 7.8 Hz), 7.47 (1H, d, ^3^*J* = 8.4 Hz), 7.56 (1H, d, ^3^*J* = 7.8 Hz), 12.21 (s, 1H, OH); *δ*_C_ (67.8 MHz, CDCl_3_) 55.2 (2C, OCH_3_), 55.3 (OCH_3_), 113.6 (6C, CH), 117.1 (C_quat_), 122.1 (CH), 129.4 (2C, CH), 129.9 (2C, CH), 130.2 (2C, CH), 131.0 (C_quat_), 132.2 (C_quat_), 132.5 (C_quat_), 134.0 (C_quat_), 134.1 (C_quat_), 134.2 (C_quat_), 136.0 (CH), 136.4 (CH), 136.8 (C_quat_), 139.5 (CH), 142.0 (C_quat_), 142.9 (C_quat_), 158.8 (C_quat_), 159.0 (2C, C_quat_), 161.0 (C_quat_), 188.0 (C_quat_, CO), 189.5 (C_quat_, CO); MS (FAB, 3-nitrobenzyl alcohol) *m/z* (%) 543 (MH^+^) (1.4). HRMS Found: 543.1805. Calcd. for C_35_H_27_O_6_: 543.1808 (MH^+^, FAB).

## 4. Conclusions

Bromoanthraquinones can be synthesized by an oxidative cycloaddition to suitably substituted naphthoquinones. Bromoanthraquinones can be reacted further to arylated anthraquinones via Suzuki-Miyaura coupling. Initial results show that also chloro substituted anthraquinones undergo Suzuki reactions in presence of the commercially available Pd(PPh_3_)_4_. The UV-VIS spectra of most of the solutions of the arylated anthraquinones in acetonitrile show at least three distinct bands associated with *π*-π* transitions. Substituent dependence of the longest wavelength transition of the three bands can be noted. 
